# Does Prophylactic Endoscopic Sclerotherapy Prevent Variceal Bleeding Or Not? A Question of Experimental Design

**DOI:** 10.1155/1994/81475

**Published:** 1994

**Authors:** Harold O. Conn

**Affiliations:** Yale University School of Medicine Department of Veterans Affairs Medical Center West Haven, Connecticut 160 Morgan Avenue East Haven, Connecticut 06512 UK


5. Ee, H. and Laurence, B. H. (1992) Haemorrhage due to erosion of a metal biliary stent through the duodenal wall. *Endoscopy*, **24**, 431–432.
6. Van Steenbergen, W., Van Aken, L. and Ponette, E. (1992) Acute pancreatitis complicating the insertion of a self-expandable biliary metal stent. *Endoscopy*, **24**, 440–442.
7. Watanapa, P. and Williamson, R. C. N. (1993) Single-loop biliary and gastric bypass for irresectable pancreatic carcinoma. *Br. J. Surg.*, **80**, 237–239.
8. Bornman, P. C., Harries-Jones, E. P., Tobias, R., van Stiegmann, G. and Terblanche, J. (1986) Prospective controlled trial of transhepatic biliary endoprosthesis *versus* bypass surgery for incurable carcinoma of head of pancreas. *Lancet*, I: 69–71.
9. Shepard, H. A., Royle, G., Ross, A. P. R., Diba, A., Arthur, M. and Colin-Jones, D. (1988) Endoscopic biliary endoprosthesis in the palliation of malignant obstruction of the distal common bile duct: a randomised trial. *Br. J. Surg.*, **75**, 1166–1168.
10. Andersen, J. R., Sørensen, S. M., Kruse, A., Rollijaer, M., Matzen, P. (1989) Randomised trial of endoscopic endoprosthesis *versus* operative bypass in malignant obstructive jaundice. *Gut*, **30**, 1132–1135.
11. Singh, S. M., Longmire, W. P. Jr. and Reber, H. A. (1990) Surgical palliation for pancreatic cancer. The UCLA experience. *Ann. Surg.*, **212**, 132–139.
12. Kahn, P. J., Skornick, Y., Inbar, M., Kaplan, O., Chaichik, S. and Rozin, R. (1990) Surgical palliation combined with synchronous therapy in pancreatic carcinoma. *Eur. J. Surg. Oncol.*, **16**, 7–11.

Prasit Watanapa
Associate Professor of Surgery
Department of Surgery
Siriraj Hospital
Mahidol University
Thailand

Robin C. N. Williamson
Professor of Surgery
Department of Surgery
Royal Postgraduate Medical School
Hammersmith Hospital
London W12 ONN

### DOES PROPHYLACTIC ENDOSCOPIC SCLEROTHERAPY PREVENT VARICEAL BLEEDING OR NOT? A QUESTION OF EXPERIMENTAL DESIGN

Some circumstantial evidence is very strong,
as when you find a trout in the milk Thoreau

**ABSTRACT**

*Van Thiel, D. H., Dindzans, V. J., Schade, R. R., Rabinovitz, M. and Gavaler, J. S. (1993) Prophylactic versus emergency sclerotherapy of large esophageal varices prior to liver transplantation. Digestive Diseases and Sciences; 38 1505–1510*

**From January 1985 through July 1987, adult patients accepted for liver transplantation with large esophageal varices were enrolled in a study evaluating the use of prophylactic vs emergency sclerotherapy. Six hundred forty-eight subjects received prophylactic sclerotherapy, and 172 received emergent sclerotherapy. Esophageal stricture formation was increased 12.9-fold ($P < 0.001$), esophageal perforation 6.4-fold ($P < 0.005$), and post-sclerotherapy bleeding esophageal ulcers 3.7-fold ($P < 0.001$) in those receiving emergency sclerotherapy as opposed to prophylactic sclerotherapy. These differences were even greater if the number of sclerotherapy sessions rather than the number of patients was used as the denominator for the comparisons. In total, 19.6% of emergency sclerotherapy cases were associated with an untoward outcome of sclerotherapy; only 1.9% of cases receiving prophylactic sclerotherapy experienced an untoward outcome ($P < 0.001$). These data demonstrate that emergency sclerotherapy is associated with a greater prevalence of complications and support earlier studies that show that sclerotherapy**

**prevents variceal bleeding over the short term. The data also suggest that when applied to patients with large varices awaiting orthotopic liver transplantation, it enhances the chance of a patient surviving to be transplanted by preventing a variceal bleed and the spiral of liver failure and death that frequently follows an episode of acute variceal bleeding.**

KEY WORDS: Sclerotherapy, portal hypertension, oesophageal varices

### PAPER DISCUSSION

Van Thiel and associates report that prophylactic endoscopic sclerotherapy (EST) in patients "with advanced liver disease awaiting liver transplantation...reduces the number of bleeding episodes, the frequency of emergency sclerotherapy sessions, and, therefore, the complications of sclerotherapy..."[1]. This conclusion appears to be at variance with most of the randomized clinical trials (RCTs) of prophylactic EST published in peer reviewed journals, which suggests that the incidence of hemorrhage from esophageal varices (HEV) and death are either unaffected[2-6] or *increased* by prophylactic sclerotherapy[7,8].

Why this difference? In my opinion it represents the difference between prospective, randomized controlled trials and poorly controlled, contrived comparisons. Both sets of observations are correct, but the two groups of patients studied were very different. Furthermore, in the transplant study of Van Thiel *et al.* the "control" group is uncontrolled, i.e., it is not an appropriate control group. Indeed, the patients who had prophylactic EST and those assigned to be the control patients are disparate groups that are vaguely defined, differently selected and, in fact, quite dissimilar.

When prophylactic EST has been evaluated in properly performed RCTs the untreated control groups have been shown to have no more fatalities and hemorrhages than those who received prophylactic EST[2-8]. In only two such studies has EST induced a significant reduction in risk[9,10], and these two investigations are the first two RCTs on prophylactic EST ever performed. They share a common flaw. In both studies many of the control patients who died of HEV did so after the second, third or some subsequent hemorrhage. In these trials the untreated control subjects were permitted to run the risks of repeated hemorrhages without the right of rescue by portal-systemic anastomosis or any other alternative form of therapy. Thus, the flaw in these studies is that they were designed before Human Investigation Committees required that "escape clauses" be incorporated into such investigations to protect patients against the failure of "control" therapy. They can be considered investigational "dinosaurs", vestiges of an earlier age of clinical research in which the primary hypotheses is relentlessly pursued unto death, if necessary. Such studies appear to be more effective in showing the hazards of an old therapy than the advantages of a new one. Furthermore, as shown in recent analyses of the quality of RCTs[11], these two early studies were awarded low quality scores. Such phenomena represent some of the disadvantages of being a pioneer and not a follower.

How did the study of Van Thiel *et al.* differ in design from the later investigations that showed that prophylactic EST seems to *increase* the risks of HEV and death? In the "negative" RCTs[2-8] the patients were *randomly* selected to have prophylactic EST or to be control subjects who were to be treated as identically as possible to the EST groups, including in one of these studies, sham EST injections of sclerosant into the esophageal lumen[8].

In the investigation of Van Thiel *et al.*[1] two groups of preselected patients, all of whom had been admitted to Presbyterian University Hospital in Pittsburgh, where they had undergone formal evaluation for liver transplantation between January 1985 and July 1987, were studied. Those who had been evaluated on the medical service had prophylactic EST "...when indicated by the size of their varices, as the attending surgeon was an advocate of EST, while those admitted to the surgical service received EST only under emergency conditions associated with active variceal hemorrhage, as the attending surgeon was skeptical of the management of potential transplant recipients by EST... No difference, other than the use of prophylactic EST on the medical service, but not the surgical service existed..." That seems to be an unambiguous statement, but is it? "*Patients were admitted to either service based solely upon the basis of the referral*" (editorialist's italics). Now that is a very ambiguous statement that could cover a multitude of biases. We learn later that "a majority of the patients with hepatocellular carcinoma and cholangiolar carcinoma were admitted to the surgical service, while more patients with advanced non-malignant, chronic liver disease were admitted to the medical service". The key phrase seems to be"... solely on the basis of referral". Therein lies the tale. Did one person make all the referrals to a single surgeon on each service? Did he or she work every hour of every day for two and one-half years? Transplant surgeons in Pittsburgh are a dedicated lot, but even Tom Starzl isn't that dedicated. Were the referrals always accepted exactly as referred, and what factors are involved in making such referrals and in their acceptance? Certainly, such factors must determine why two-thirds of the patients accepted for possible liver transplantation were on the surgical service and only one-third on the medical service. Somehow more than half the patients with large varices (686) were on the medical service, compared to only 628 on the surgical service. Furthermore, the odds of dying of HEV on the surgical service was 6.1 times greater than on the medical service. It seems unlikely that prophylactic EST was performed in equal numbers of medical and surgical patients. Somehow, other factors than the spontaneous occurrences of HEV, which excluded patients from prophylactic EST, must have been operative.

Thus, the authors' conclusions cannot be accepted as presented because the groups compared are not comparable.

Somehow, the assignment of the two groups of patients to the medical and surgical services reminds me of a conversation between two pediatric patients. The veteran patient, who awoke one morning to find a new patient lying in the next bed asked, "Are you medical or surgical?"

The newcomer responded, "How can you tell?"

The first patient replied, "Medical patients come to the hospital sick and get well; surgical patients are admitted healthy and get sick." Perhaps, the study of Van Thiel is just another example of that syndrome.

Finally, I believe that if a RCT evaluating EST is ever performed in unselected patients awaiting transplantation, it will show that the patients who require emergency EST will have more complications of EST than those who do not, as was found by Van Thiel *et al.* and as would be expected. I predict, however, that those who had prophylactic EST will probably also have more endoscopic complications and, probably, a higher mortality rate than those who did not have prophylactic EST. It is clear that I have strong faith in real RCTs!

### ADDENDUM

Prior to submitting this manuscript to *HPB Surgery* I sent a copy of it to Dr. Van Thiel and requested his criticisms and suggestions. He responded as follows:

Dear Dr. Conn,

I think you missed several critical points. These are:

1. Unlike all other studies comparing prophylactic sclerotherapy vs. no treatment, our study and its conclusions are limited to patients with advanced disease referred to and waiting for a liver transplant. Survival was to time of transplant not until death. We simply say that in this high risk group, elective prophylactic therapy is safer than emergent therapy. Moreover, it en-ables more patients to remain active transplant candidates and ultimately to survive to be transplanted.
2. The fact that the patients who were sicker were on the medical service and more tumor patients were on the surgical service does mean the groups were different but would not have been expected to bias the results in favor of those on the transplant service (less time to transplant for tumor patients; less severe hepatic disease; fewer cases actually receiving the potentially dangerous sclerotherapy). This, however, did not occur.

With these two exceptions and the recognition that doing studies of this sort in a transplant population is difficult at best. I have to agree with your other comments. Nonetheless, our study has value and one can learn from an experience that is not randomized. Most of us learn, most of what we learn about life, let alone medicine, in an uncontrolled manner. Yet we learn.

David van Thiel, M. D.

I agree that one can learn from nonrandomized observations, but not when the questions are begged and the results are loosely interpreted.

Harold O. Conn, M. D.
Professor of Medicine, Emeritus
Yale University School of Medicine
Department of Veterans Affairs Medical Center,
West Haven, Connecticut
160 Morgan Avenue
East Haven, Connecticut 06512

### REFERENCES

1. Van Thiel, D. H., Dindzans, V. J., Schade, R.R., Rabinovitz, M., Gavialer, J. S. (1993) Prophylactic versus emergency sclerotherapy of large esophageal varices prior to liver transplantation. *Dig. Dis. Sci.*, **38**, 1505–1510.
2. Koch, H., Henning, H., Grimm, H., Soehendra, N. (1986) Prophylactic Sclerosing of esophageal varices: results of a prospective controlled study. *Endoscopy*, **18**, 40–43.
3. Kobe, V. E., Schentke, K. U. (1987) Endoskopische sklerotherapy (EST) von osophagusvarizon studien zur teknik und zum krankheitsverlauf. Dtsch z Verdau Stoffwechsel Krankh, **47**, 151–157.
4. Sauerbruch, T., Wotzka, R., Kopke, W., Harlin, M., Heldwein, W., Bayerdorffer, E., Sander, R., Ansari, H., Starz, I., Paumgartner, G. (1988) Prophylactic sclerotherapy before the first episode of variceal hemorrhage in patients with cirrhosis. *N. Engl. J. Med.*, **319**, 8–14.
5. Fleig, W. E., Stange, E. F., Wordehoff, D., Preclik, G., Nuber, R., Rainer, K., Ditschuneit, H. (1988) A randomized trial comparing prophylactic (PS) and therapeutic sclerotherapy in cirrhotic patients with large esophageal varices and no previous hemorrhage (abstr). *J. Hepatol.*, 7 (Suppl): S128.
6. De Franchis, R., Primignani, M., Arcidiacono, P. G., Rizzi, P. M., Vitagliano, P., Vassoler, M. C., Arcidiacono, R. *et al.* (1991) Prophylactic sclerotherapy in high-risk cirrhotics selected by endoscopic criteria. Gastroenterology, **101**, 1087–1093.
7. Santangelo, W. C., Dueno, M. I., Estes, B. L., Krejs, G. J. (1988) Prophylatic sclerotherapy of large esophageal varices. *N. Engl. J. Med.*, **316**, 814–818.
8. Veterans Affairs Cooperative Variceal Sclerotherapy Group. (1991) Prophylactic sclerotherapy for esophageal varices in men with alcoholic liver disease. *N. Engl. J. Med.*, **324**, 1779–1784.
9. Paquet, K. J. (1982) Prophylactic endoscopic sclerosing treatment of the esophageal wall in varices – a prospective controlled randomized trial. *Endoscopy*, **14**, 4–5.
10. Witzel, L., Wolbergs, E., Merki, H. (1985) Prophylactic endoscopic sclerotherapy of oesophageal varices: a prospective controlled study. *Lancet*, **1**, 773–775.
11. Pagliaro, L., D'Amico, G., Sorensen, T. I. A. (1992) Prevention of first bleeding in cirrhosis. A meta-analysis of randomized trials of nonsurgical treatment. *Ann. Int. Med.*, **117**, 59–70.

